# Parental Knowledge of Appendicitis and Preference for Operative or Non-Operative Treatment at a United Kingdom Children’s Hospital

**DOI:** 10.3390/children9081191

**Published:** 2022-08-09

**Authors:** Kitty Monks, Nigel J. Hall

**Affiliations:** 1 University Surgery Unit, Faculty of Medicine, University of Southampton, Southampton SO16 6YD, UK; 2 Department of Paediatric Surgery and Urology, Southampton Children’s Hospital, Southampton SO16 6YD, UK

**Keywords:** appendicitis, children, parental knowledge, patient opinion, treatment preference

## Abstract

Appendicitis is a common cause of abdominal pain in children, and is the most common reason for children to undergo emergency abdominal surgery. To guide our research program in this field, we aimed to determine parental understanding with regard to appendicitis and its treatment. We also wished to assess parental preference for non-operative or surgical treatment of children with uncomplicated appendicitis. We asked parents attending a children’s outpatient clinic to complete a brief questionnaire that was interspersed with educational content to rectify any knowledge gaps. A total of 396 parents (of 414 approached) agreed to participate. There were gaps in parental knowledge, including not knowing where the appendix is located (one-third of respondents), not knowing what appendicitis is (40% of respondents), and underestimating the incidence of appendicitis. Parents typically overestimated the risks of complications and dying from both uncomplicated and complicated appendicitis. There was no influence of gender, age, or previous experience of appendicitis on these findings. When presented with the scenario of equal effectiveness of non-operative treatment and surgery for uncomplicated appendicitis, 59% of respondents expressed a preference for non-operative treatment over surgery, while 21% expressed a preference for surgery (20% expressed no preference). These findings are important for clinicians and researchers when discussing appendicitis and treatment options with families, and justify ongoing research into the comparative effectiveness of non-operative treatment and surgery.

## 1. Introduction

Acute appendicitis is a common cause of abdominal pain in children, and is the most common reason for their emergency admission to general surgical units. In the UK, approximately 10,000 cases in children are treated annually [1]. Appendicitis is most prevalent in those between the ages of 10 and 20 years, and the lifetime risk of developing appendicitis is approximately 8% [2]. Despite many decades of investigation, there remain uncertainties regarding the optimal treatment of a number of aspects of appendicitis in children. These include the optimal method of diagnosis [3], the role of diagnostic imaging [4,5], non-operative treatment compared to surgery [6,7,8], postoperative antibiotic type and duration [9], and the optimal ways to treat complications following surgery, such as deep surgical site infection or prolonged ileus [4]. Our program of research aims to investigate a number of these areas.

In paediatrics, parent/guardian (henceforth referred to simply as parents) involvement is crucial for the optimal care of children. Parents have the responsibility of making decisions about their children’s health and wellbeing. It is therefore essential that they are able to make informed and appropriate decisions about the health and welfare of their children. Parents also have influence over the decisions children make about their own healthcare [10,11]. To make these decisions, parents require sufficient information and knowledge to help them in their decision-making process. An understanding of parental knowledge is therefore important in guiding the provision of information. An understanding of parental knowledge and understanding is also important for researchers when designing clinical research studies involving children and their parents. Only by fully appreciating the knowledge, understanding, and attitudes of parents can we design appropriate research studies that seek their involvement.

In order to inform our research program, we sought to ascertain the current knowledge and understanding of parents with regard to appendicitis. In addition, given our current interest in the role of non-operative management of appendicitis as an alternative to surgery [6,12,13], we investigated parents’ attitudes towards non-operative treatment of acute appendicitis.

## 2. Materials and Methods

A questionnaire was developed that would enable us to ascertain the knowledge and understanding of parents with regard to appendicitis and its treatment, along with their prior experience of the illness, and their attitudes towards non-operative and operative treatment. The questionnaire was based on those used in previous similar studies outside the UK [10,11,12], and the questions were interspersed with short education sections to rectify any knowledge gaps. Having created a draft of the questionnaire, we sought input from a group of young people and parents to provide patient and public involvement (PPI) in our research program. This enabled us to assess the questionnaire for content, readability, understanding, and through cognitive interviews with some of the group for measurement/observation error. Following this, we modified questions that were unclear or ambiguous to ensure that the responses provided accurately reflected the answers that the participants intended. This enabled us to ensure that the questionnaire was as accessible as possible to the participants, and that it used terms that were easy to understand (e.g. we used the terms “simple” and “perforated/burst” to describe uncomplicated and complicated appendicitis, respectively). The questionnaire was designed to be completed on an electronic tablet device using a commercially available survey platform (iSurvey), with a printed paper alternative available.

Parents (aged 16 years or over) of children (aged 16 years or younger) attending the general paediatric outpatient department of our hospital were approached by a single medical student (K.M.) and asked to complete the questionnaire whilst waiting for their child’s outpatient appointment. The age of 16 years was used because this is the upper age limit for children seen in our hospital’s outpatient department. Written consent to participate in the study was provided. Respondents could complete the questionnaire on either an electronic tablet or paper. The questionnaire (Appendix SA) took approximately 10 min to complete.

A convenience sample of participants attending the outpatient department over a 2-month period starting in October 2018 was used. We aimed to obtain a minimum of 200 responses.

Electronic responses were exported directly from the online survey platform into Microsoft Excel, and paper responses were added manually. Prior to analysis, we predetermined that we would describe the data descriptively, but also plan subgroup analyses driven by our current research interests. These included the impacts of gender, age, and prior knowledge of someone with appendicitis on the survey responses—particularly with regard to understanding about appendicitis’ treatment and risks, as well as treatment preferences. Statistical analyses were performed using IBM SPSS Statistics for Windows, version 28.1 (IBM Corp., Armonk, NY, USA). All responses were included in the analysis as long as at least one of the questionnaire questions had been answered (if only the demographic details section had been completed then the response was excluded).

The study was approved by the East Midlands Research Ethics Committee in September 2017 (ref 17/EM/0344), as well as the ethics committee of the University of Southampton Faculty of Medicine. No specific funding was used for the conduct of the study.

## 3. Results

### 3.1. Respondents

During the study period a total of 396 parents completed a questionnaire; all were included. A total of 18 individuals who were approached declined to participate. A full set of responses is provided in the Supplementary Materials; the key findings are summarised here. Of the respondents, 82 were male, and 294 were female (20 did not state). The respondents’ age ranged from 16 years to >64 years, and the age of the oldest child ranged from 1 month to 44 years. The highest level of education achieved by respondents was GCSE/O level (i.e., completion of secondary education to age 16) in 65, apprenticeship or similar in 35, A level (i.e., completion of education to age 18) in 93, and university degree or higher in 173 (30 unknown). The majority of respondents (253/387, 65%) knew someone who had been treated for acute appendicitis, whereas 33% (127/387) did not, with the remaining 7 responding “don’t know”. Of those who did know someone who had been treated for appendicitis, the relationships to them were as shown in Table 1.

### 3.2. Knowledge and Understanding of Appendicitis

**Figure 1:**
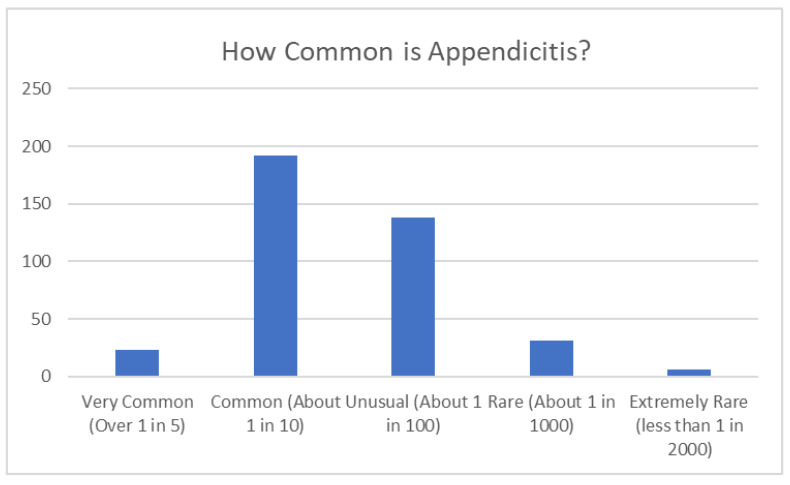
Respondents’ understanding regarding the frequency of developing appendicitis.

**Figure 2:**
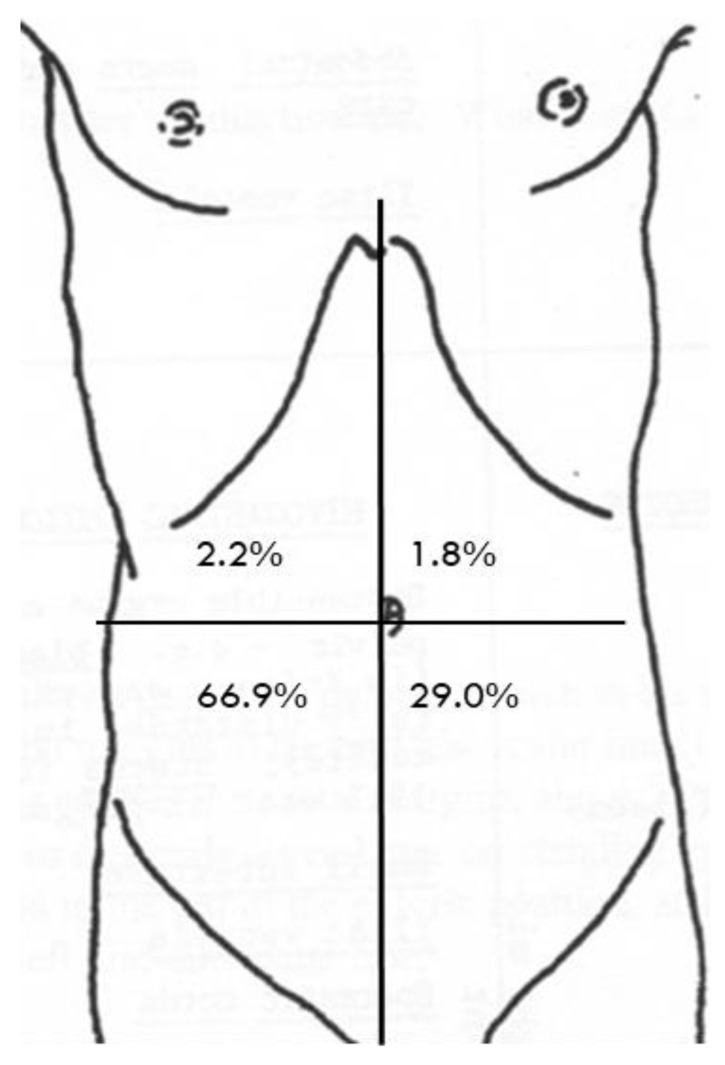
Respondents’ understanding regarding the location of the appendix in the abdomen.

The most frequent response (49%) to the question about frequency of appendicitis was “Common—about 1 in 10”, with the distribution shown in Figure 1, and most respondents (67%) were able to correctly identify the site of the appendix within the lower-right quadrant, although nearly one-third chose the lower-left quadrant (Figure 2). The most common keywords extracted from the free text descriptions regarding respondent understanding of appendicitis were inflammation and/or infection (in 57%). All keywords extracted are shown in Supplementary Table S1. The experiences of the individuals who the respondents knew who had had appendicitis were described as shown in Table 2.

### 3.3. Understanding of Treatment and Risks of Appendicitis

The most frequent response provided about current usual treatment for appendicitis was surgery (348/387; 90%), with just under 9% (34/387) responding antibiotics alone, and 1% answering pain relief and bed rest. When asked how likely it was that a child with appendicitis would suffer from a burst appendix if they did not have surgery straight away, over three-quarters responded either “likely” or “very likely” (Table 3). Over 90% of respondents thought it “likely” or “very likely” that a child would become “very sick or even die” as soon as their appendix burst. Only 9% thought it “unlikely” or “very unlikely”.

Serious complications were thought to occur with a frequency of 1 in 4 or more frequently following simple appendicitis by 30% of respondents, and following perforated appendicitis by 73%. Death was thought to occur at a frequency of 1 in 4 or more frequent by 6% of respondents, and following perforated appendicitis by 31%. 

There were no statistically significant relationships between respondents who knew someone who had previously had appendicitis and the frequency with which they thought that appendicitis occurred, nor the perceived frequency of significant complications or death from either simple or complicated appendicitis. However, those who knew someone who had previously had appendicitis more frequently identified the correct incidence (“about 1 in 10”) of appendicitis (52% vs. 40%; Supplementary Figure S1).

### 3.4. Opinions Regarding Different Treatments for Appendicitis

In total, 306 of the 380 respondents (81%) indicated that they would probably or definitely consider their child being treated with antibiotics alone if they were to develop simple appendicitis, compared to 44% (167/377) who would probably or definitely consider surgery. Overall, there was a preference for antibiotics over surgery for 59% (223/375), a preference for surgery over antibiotics for 21% (78/275), and no preference for 20% (78/375). No significant associations were identified between the age or gender of the respondent, or their previous experience (knowing someone) with appendicitis, and their treatment preference. However, there was a significant (*p* = 0.004) association between higher educational attainment and treatment preference, such that a greater level of education was associated with a preference for non-operative treatment.

## 4. Discussion

In this study, we aimed to document parental understanding about appendicitis, along with its risks and treatment, and to determine attitudes to operative and non-operative treatment of uncomplicated appendicitis. The rationale for the study was to inform the design of our ongoing research program and, in particular, to guide the provision of information to parents through discussions and information leaflets. While previous similar work in this field has been undertaken, to the best of our knowledge this is the first such study to be completed in the UK. This is important, as there may be differences in knowledge and attitudes based on culture. Overall, we identified some knowledge gaps in understanding about appendicitis, some misunderstandings about the risks of appendicitis, and that whilst both non-operative treatment with antibiotics and surgery are accepted treatments, there appears to be a preference for non-operative treatment if both are equally effective.

Two-thirds of respondents (67%) were able to correctly identify the location of the appendix in the lower-right quadrant of the abdomen, and just over half of respondents (57%) included the keywords infection and/or inflammation in their responses about their understanding of what appendicitis is. These findings indicate some gaps in parental knowledge that clinicians and researchers may wish to take into account when providing written and/or verbal information. Additionally, these findings may have clinical implications—a lack of awareness regarding the correct location of the appendix may mean that parents of a child with worsening lower-right-quadrant pain are unaware of the possibility of appendicitis and, therefore, do not present to hospital. Improving this knowledge—for example, via public health interventions—may reduce late presentations of appendicitis. Despite this, the majority of respondents (90%) were aware that the typical treatment of appendicitis is appendectomy.

When considering respondents’ awareness of the risks of appendicitis, three-quarters believed that it was likely or very likely that the appendix would burst if surgery was not performed immediately, and 90% believed that it was likely or very likely that a child would become very sick or even die were the appendix to burst. Similarly, when considering the risks of serious complications and death, we found that parents typically overestimated the frequency at which serious complications or death occur following both simple and perforated appendicitis, although more so for perforated disease. Knowing that parents typically overestimate the risks associated with appendicitis provides a useful understanding of the context for clinicians and researchers when having discussions with families about treatment and research. Clinicians may find it helpful to reassure parents that risks may be present yet perhaps not as high as anticipated. Researchers may find it useful to address these risks during discussions about research, particularly if parents consider that involvement in research may increase the risks to their child.

A small number of previous similar studies have investigated knowledge about appendicitis and its treatment, as well as attitudes towards non-operative treatment of appendicitis, with mixed observations [14,15,16,17,18]. To the best of our knowledge, no previous investigation has been undertaken in the UK, with the majority conducted in the USA. Kadera et al. identified similar knowledge gaps to those that we observed, similar misunderstandings regarding the risks of appendicitis, and also a preference for non-operative over surgical treatment. Their study involved 129 adults at a single hospital in the USA. In a single-centre study involving parents from the USA responding in relation to appendicitis in their child, similar knowledge gaps existed to those that we encountered [15]. In a larger study from the USA, Hanson et al. surveyed over 1700 individuals [16], of whom nearly 80% expressed a preference for laparoscopic appendectomy for their child were they to develop uncomplicated appendicitis. Their sample had a strong medical influence, since the survey was advertised at a medical school campus and through the contacts of the investigative team. In a smaller subsample (*n* = 220) recruited at public (as opposed to medical) venues, non-operative treatment was viewed more favourably. Althans et al. surveyed 255 medical students in a single medical school in the USA, and presented them with realistic risks regarding the efficacy of non-operative treatment and adverse events related to both surgery and antibiotic therapy. They reasoned that medical students were in a unique position of being potential patients with greater medical knowledge than the general public and, possibly, less bias than practicing physicians. Two-thirds of respondents expressed a preference for surgical treatment, while 24% expressed a preference for non-operative treatment [18].

Outside of the USA, Bom et al. reported the findings of a study conducted by an independent market research agency in the Netherlands [17]. They hoped to remove the influence of pre-existing medical knowledge through this independent methodology, and found that a similar proportion of adults expressed a preference for non-operative treatment (49%) and for surgery (44%). In a recently reported study similar to ours, Ensor et al. surveyed 311 parents attending an outpatient clinic at a specialist children’s hospital in Australia [19]. They also found that parents typically overestimated the risk of dying from perforated appendicitis, and found similar proportions preferring non-operative (52%) and surgical treatments (48%).

There are some limitations to our study, which should be considered when interpreting our findings. Although we had a relatively large sample size, there was an imbalance between male and female respondents, which may have influenced our results overall. This is a consequence of our approach of using a convenience sample rather than purposively sampling to achieve equal gender distribution. However, in subgroup analyses for each key metric, there were no statistically significant differences between male and female respondents. A further limitation is that the study was completed in a single hospital. It is possible that there may be geographic variation in knowledge and understanding about appendicitis that we have not been able to detect. Despite these limitations, we believe that our large sample size provides an extremely useful overview of UK parents’ knowledge, understanding, and opinion. A limitation regarding the questions about non-operative treatment is that we set the context of these questions as being that non-operative treatment and surgery are equally effective (although we did not further define effectiveness). This suggestion was made by our PPI group, given that providing adequate estimates of the comparative effectiveness of these two treatment modalities based on current evidence is unclear, and research is ongoing. Despite this, our findings support ongoing investigation of the comparative effectiveness of non-operative treatment and surgery. Whether a preference for non-operative treatment remains once comparative data are available remains to be seen. As with any survey, hypothetical choices made by parents may differ from the choices they may make if their child were to actually develop appendicitis, yet our observations in treating patients and through our research confirm significant interest in non-operative treatment [13,20]. We should also note that this work was undertaken prior to the SARS-CoV-2 pandemic, a period during which there was much greater use of non-operative treatment than had previously been the case—certainly within the UK [21]. Whether attitudes have changed as a result is uncertain.

## 5. Conclusions

In conclusion, we found that knowledge gaps exist in parental understanding of appendicitis and its treatment, and that parents generally overestimate the risks of appendicitis. These findings are similar to others reported outside the UK. Our data suggest that there is significant interest in non-operative treatment, and that there is a preference for it over surgery if they are equally effective. This knowledge is useful when discussing appendicitis and its treatment with families, and when designing future research in this field.

## Figures and Tables

**Table 1:** Relationship to respondent in those who knew someone who had had appendicitis.

Who?	Number of Responses (*n* = 251)
Myself	37 (14.7%)
My child	13 (5.2%)
My parent/sibling	51 (20.3%)
Other family member	53 (21.1%)
A friend	97 (38.6%)

**Table 2:** Rated experience of appendicitis in those who knew someone who had had appendicitis.

Experience of Symptoms and Treatment	Number of Responses (*n* = 251)
Very easy	6 (2.3%)
Easy	47 (18.7%)
Neither easy nor difficult	75 (29.8%)
Difficult	87 (34.7%)
Very difficult	36 (14.3%)

**Table 3:** Perceived likelihood of a child with appendicitis suffering from a burst appendix if they did not proceed to surgery straight away.

Likelihood	Number of Responses (*n* = 386)
Very likely	95 (24.6%)
Likely	223 (57.8%)
Unlikely	56 (14.5%)
Very unlikely	12 (3.1%)

## Data Availability

Anonymised data will be made available by the study team upon reasonable request so long as the request is accompanied by a protocol outlining the proposed use of the data and confirmation of ethical approval from the requesting institution. A fully executed data sharing agreement will also be required.

## Supplementary Materials

The following supporting information can be downloaded at: https://www.mdpi.com/article/10.3390/children9081191/s1, Table S1: Keywords extracted from free text responses of respondents’ understanding of appendicitis. Figure S1: Respondents’ understanding of the frequency of appendicitis based on previously knowing someone who had been treated for appendicitis. Appendix SA: Assessing Parental Knowledge and Views on Appendicitis and its Treatment.
