# Supplementary material for: Parental Knowledge of Appendicitis and Preference for Operative or Non-Operative Treatment at a United Kingdom Children’s Hospital
Source: Children (Basel). 2022 Aug 9;9(8):1191. doi: 10.3390/children9081191 (PMC9406866; doi:10.3390/children9081191)
Supplement: Supplementary file 1 [file children-09-01191-s001.zip › Appendix SA.pdf]

Assessing Parental Knowledge and Views on Appendicitis and its Treatment

Questionnaire for parents: version 2.0 September 6th 2017

We are currently doing research into different types of treatment for appendicitis. To help us with our research we are interested in finding out what parents *already know and understand about appendicitis* and how it is treated.

Regardless of whether you or your child have any experience of appendicitis, we would be grateful if you can spare a few minutes of your time to answer the questions below.

Thank you.

1. Introductory statement

Involvement in this project simply involves completing this questionnaire. It should take no longer than about 5 minutes. It is entirely optional. We are really grateful for your opinions which will be extremely helpful to us. There are no specific benefits to you from taking part but no risks either. All information you provide will be anonymous and cannot be withdrawn once you submit it.

This project has been approved by the Leicester Central NHS Research Ethics Committee.

Should you have any questions about this project at any stage please feel free to email Mr Nigel Hall, Consultant Paediatric Surgeon, the study supervisor at n.j.hall@soton.ac.uk
In the unlikely event of concern or complaint, please contact the University of Southampton Research Governance Manager (02380 595058, rgoinfo@soton.ac.uk).

In completing this questionnaire I understand the above and agree for the information I provide to be used for the purposes of this project. I understand that all information I provide will be kept anonymous and that my answers cannot be withdrawn once submitted.

Please initial and date here: ____________________

2. What you understand about appendicitis:

1. What do you understand by the term appendicitis?

____

2. How common do you think appendicitis is in the UK? This means the chance of developing appendicitis at any stage in a person's life. (Please circle your choice)

Very common (over 1 in 5)	Common (about 1 in 10)	Unusual (About 1 in 100),	Rare (About 1 in 1000)	Extremely rare (Less than 1 in 2000)

3. **Where in the body would you expect to find the appendix?**

   Please mark with an X

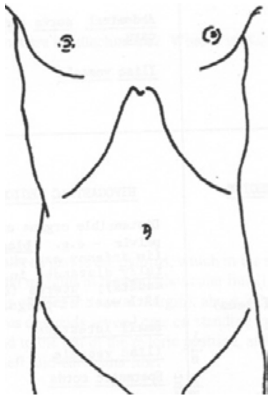

Appendicitis is the swelling of the appendix due to obstruction, leading to infection and inflammation. This leads to loss of appetite, nausea and vomiting. The characteristic pain of appendicitis is a dull pain that begins by the tummy button and then moves and becomes a very severe pain in the lower right hand side of the stomach.
Appendicitis occurs in 7-8% people during their lifetime.

4. **Do you know someone that has been treated for appendicitis?**

Yes / No / Don't know

If you answered No or Don't know please move to Question 7

5. **If yes who?**

Myself	My child	My parent or sibling	Other family member	A friend

6. **Please rate their experience of their symptoms and treatment + any complications if they had them**

Very easy	Easy	Neither easy nor difficult	Difficult	Very difficult

7. **Based on your understanding, what is the current usual treatment for appendicitis?**

   a. Pain relief and bed rest ☐
   b. Treatment just with antibiotics ☐
   c. Surgery (an operation to remove the appendix) ☐

8. **Please rate this statement: if a child has appendicitis and they don't have surgery straight away, how likely do you think it is for the appendix to burst (perforate)?**

Very unlikely	Unlikely	Likely	Very likely

9. **Please rate this statement: as soon as an appendix bursts the child will become very sick – and may even die.**

Very unlikely	Unlikely	Likely	Very likely

There are two main types of appendicitis.
It can be SIMPLE, this is where there is the swelling of the appendix, leading to pain and other symptoms, but without rupture of the structure of the appendix.
In PERFORATED appendicitis, the appendix bursts, spilling out the contents into the rest of the abdominal cavity; this can be more serious.

10. In children with SIMPLE (not burst/perforated) appendicitis:

(a) How likely are they to develop serious complications from appendicitis?

Extremely rarely (under one in 1000)	Rarely (under one in 100)	Unlikely (about one in 10)	Often (about one in four)	Usually (over one in two	Nearly always

(b) How likely are they to die from appendicitis?

Extremely rarely (under one in 1000)	Rarely (under one in 100)	Unlikely (about one in 10)	Often (about one in four)	Usually (over one in two	Nearly always

11. In children with PERFORATED/BURST appendicitis:

(a) How likely are they to develop serious complications from appendicitis?

Extremely rarely (under one in 1000)	Rarely (under one in 100)	Unlikely (about one in 10)	Often (about one in four)	Usually (over one in two	Nearly always

(b) How likely are they to die from appendicitis?

Extremely rarely (under one in 1000)	Rarely (under one in 100)	Unlikely (about one in 10)	Often (about one in four)	Usually (over one in two	Nearly always

The main complications of appendicitis are perforation of the appendix and infection inside the abdomen. The risk of death from appendicitis in the UK is very low; 0.08% for simple appendicitis, and 0.5% for perforated appendicitis.

Previously surgeons believed that any patient with appendicitis should have immediate surgery (within an hour). We now know that urgent surgery (within a day) is safe.

This has prompted research into other treatments for appendicitis including treatment with just antibiotics and no surgery at all.

We are currently studying the use of antibiotics alone as the main treatment for SIMPLE appendicitis in children and an alternative to surgery. This has the advantage of avoiding having a general anaesthetic and an operation. Studies have shown that the majority of children with SIMPLE appendicitis can safely and effectively be treated with antibiotics alone without needing an operation

12. **If it were up to you, how would you feel about your child having the following treatments for SIMPLE appendicitis, assuming that they were equally effective?**

A – treatment with antibiotics (initially in hospital and then at home) with surgery only if the appendicitis happened again

Definitely yes	Probably yes	No preference	Probably not	Absolutely not

B – surgery straight away to remove the appendix

Definitely yes	Probably yes	No preference	Probably not	Absolutely not

<u>General Questions</u> – it would be really helpful if you could also complete these more general questions please. This will help us to understand if different people, perhaps of different ages or from different backgrounds have different understanding about appendicitis or different views about treatment options:

1. **What is your highest level of education?**
   a. O-Levels/GCSE
   b. A-Levels/BTEC
   c. Apprenticeship or similar
   d. University degree (BA / BSc or equivalent)
   e. Postgraduate (Masters/PhD or equivalent)

2. **Are you: Male / Female**

2. **What is the first part of your postcode?**

   ____

3. **How old are you?**
   16-24 / 25-34 / 35-44 / 45-54 / 55-64 / >64

4. **How old is you oldest child?**

   __________ (yrs)

If your child has previously had appendicitis and you are interested in helping us with further research in this area please tick this box and provide your email address and we will contact you. ☐

If there is anything in this questionnaire that you would like to discuss with one of the researchers please tick this box and provide your email address here and we will contact you. ☐

If you would like to be informed of the results of the study once they are available please tick this box and provide and email address. ☐

Email address: ____

You do not have to provide an email address, please only do so for one of the reasons above. If you provide your email address we will only use it for the purpose you have indicated above. We will not share it with anyone else. We will delete your email address either once we have contacted you or after 6 months, whichever is sooner.

THANK YOU FOR COMPLETEING THIS QUESTIONNAIRE
