# Supplementary material for: Parental Knowledge of Appendicitis and Preference for Operative or Non-Operative Treatment at a United Kingdom Children’s Hospital
Source: Children (Basel). 2022 Aug 9;9(8):1191. doi: 10.3390/children9081191 (PMC9406866; doi:10.3390/children9081191)

Keyword	Number of Responses<br><br>= 396
Burst and removal	1
Inflammation	88
Pain	41
Confirmation of Knowledge	7
Burst/rupture	32
Infection	51
Inflammation and infection	35
Inflammation and pain	13
Position/use of appendix	8
Infection and pain	8
Inflammation and rupture	20
Inflammation and position	1
Unsure/no knowledge	32
Pain that can become dangerous	5
Inflammation and removal	4
Infection and rupture	7
Requires emergency surgery	1
Problem/complication of appendix	12
Pain that may lead to removal	4
Leading to poisoning	1
Appendix has no use	4
Burst appendix can have serious complications	8
Importance of assessment	1
Mention of surgery	4
Condition that requires medical attention	1
Release of dangerous toxins	2
Intestine stone	1
Irritated organ	1
Medical condition	3

Table S1: Keywords extracted from free text responses of respondents understanding of appendicitis.

Figure S1. Respondents understanding of frequency of appendicitis based on previously knowing someone who had been treaetd for appendicitis

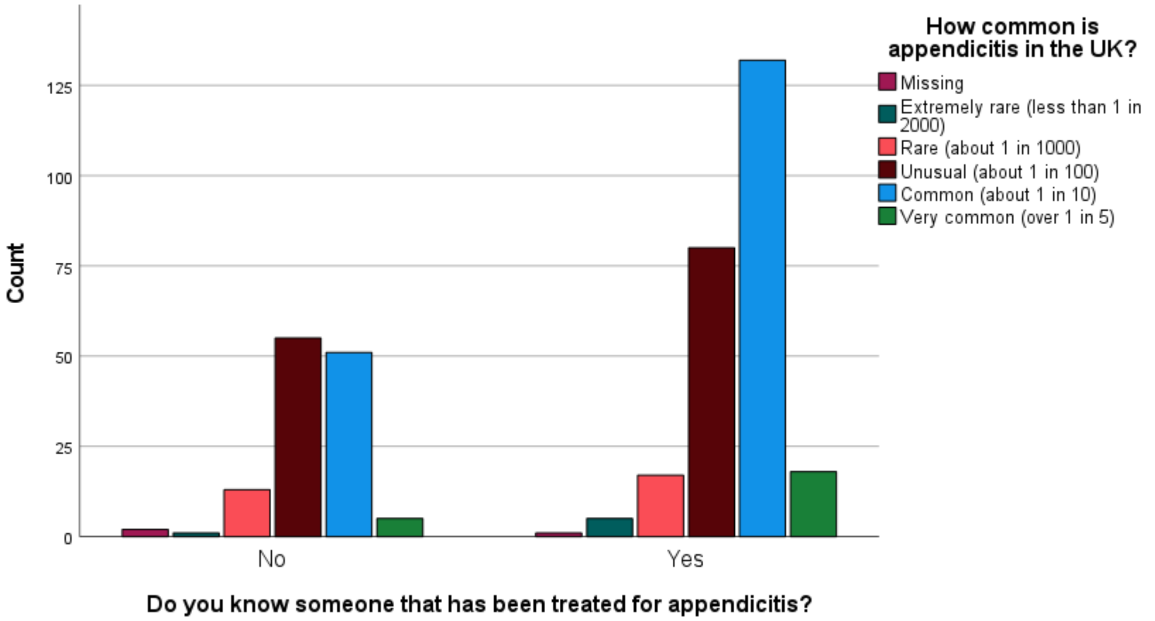

Supplement: Supplementary file 1 [file children-09-01191-s001.zip › Table S1 and Figure S1.pdf]
